# Critical Hierarchical Appraisal and Reporting Tool for Composite Measures (CHAOS)

**DOI:** 10.7759/cureus.36210

**Published:** 2023-03-15

**Authors:** Yi-Sheng Chao, Chao-Jung Wu, Hsing-Chien Wu, Hui-Ting Hsu, Yen-Po Cheng, Yi-Chun Lai, Wei-Chih Chen

**Affiliations:** 1 Syndrome Mining/Index Mining/Research, Independent Researcher, Montreal, CAN; 2 Computer Sciences, Université du Québec à Montréal, Montreal, CAN; 3 Internal Medicine, National Taiwan University Hospital Jinshan Branch, New Taipei City, TWN; 4 Pathology, Changhua Christian Hospital, Changhua City, TWN; 5 Neurological Surgery, Changhua Christian Hospital, Changhua City, TWN; 6 Chest Medicine, National Yang Ming Chiao Tung University Hospital, Yilan, TWN; 7 Chest Medicine, Taipei Veterans General Hospital, Taipei, TWN

**Keywords:** composite measures, indices, hierarchical assessment, reporting, critical appraisal

## Abstract

Background

Composite measures are often used to represent certain concepts that cannot be measured with single variables and can be used as diagnoses, prognostic factors, or outcomes in clinical or health research. For example, frailty is a diagnosis confirmed based on the number of age-related symptoms and has been used to predict major health outcomes. However, undeclared assumptions and problems are prevalent among composite measures. Thus, we aim to propose a reporting guide and an appraisal tool for identifying these assumptions and problems.

Methods

We developed this reporting and assessment tool based on evidence and the consensus of experts pioneering research on index mining and syndrome mining. We designed a development framework for composite measures and then tested and revised it based on several composite measures commonly used in medical research, such as frailty, body mass index (BMI), mental illness diagnoses, and innovative indices mined for mortality prediction. We extracted review questions and reporting items from various issues identified by the development framework. This panel reviewed the identified issues, considered other aspects that might have been neglected in previous studies, and reached a consensus on the questions to be used by the reporting and assessment tool.

Results

We selected 19 questions in seven domains for reporting or critical assessment. Each domain contains review questions for authors and readers to critically evaluate the interpretability and validity of composite measures, which include candidate variable selection, variable inclusion and assumption declaration, data processing, weighting scheme, methods to aggregate information, composite measure interpretation and justification, and recommendations on the use.

Conclusions

For all seven domains, interpretability is central with respect to composite measures. Variable inclusion and assumptions are important clues to show the connection between composite measures and their theories. This tool can help researchers and readers understand the appropriateness of composite measures by exploring various issues. We recommend using this Critical Hierarchical Appraisal and repOrting tool for composite measureS (CHAOS) along with other critical appraisal tools to evaluate study design or risk of bias.

## Introduction

Currently, there is no critical appraisal tool specifically designed for innovative composite measures. Composite measures are often derived from multiple variables and can be used as diagnoses, prognostic factors, or outcomes in clinical and health research [1,2]. For example, frailty is a diagnosis that can be confirmed based on the number of age-related symptoms and has been used to predict major health outcomes, such as mortality and falls [1]. The Charlson Comorbidity Index is another example and uses patients’ ages and comorbidities to calculate points that can be converted to the probabilities of survival in 10 years [2]. Composite measures aggregate information from multiple variables to represent certain concepts that are often difficult to quantify with single variables [1,2]. Indices, scales, and typologies are different forms of composite measures designed for specific contexts [3]. Composite measures, including indices and diagnoses, are important for representing concepts and creating proxy measures in medical research [1,2,4]. One widely used approach is to create an index by summing the variables that are scaled to similar ranges [5,6]. Disease status can be confirmed with ratings exceeding prespecified thresholds [1,2,7,8]. For example, one method used to diagnose frailty sums 70 variables, each of which ranges from 0 to 1, representing the severity of age-related symptoms [1,5]. Individuals who have a sum of symptoms exceeding a threshold are diagnosed with frailty [1,5]. Another example is the diagnosis of mental illnesses that is often based on symptoms and is confirmed by complex diagnostic criteria, such as major depressive disorder, dysthymic disorder, and bipolar disorder [4,9]. The complex diagnostic criteria may involve censoring, categorizing, and transforming input symptoms [4]. The other example is the body mass index (BMI), a measure of obesity, derived by dividing weight in kilograms by the square of height in meters [1]. Individuals can be categorized further into different weight statuses based on BMI [10]. In 2017, a composite measure development framework was published to help researchers or clinicians develop their own indices or composite measures [4].

However, several composite measures have been found to be problematic for various reasons, including a lack of evidence-based data processing, arbitrary and implicit assumptions, a poor assessment of derived measures [1,5,6], and insufficient evidence to guide variable selection [7]. The creation of composite measures by summing multiple variables is based on several implicit assumptions that may not be supported by evidence [1,8]. For example, an assumption is often made that input variables are similarly important and thus should be given the same weight [1,2]. Based on some frailty models, frailty symptoms are considered of the same importance, and the number of frailty symptoms is a proxy measure of frailty severity [1]. Another assumption is that input variables can be linked to each other, especially when they are used for outcome prediction [1,9,10]. The relationships or correlations between input variables are not routinely examined before mining these indices or creating novel medical diagnoses [4,6]. Researchers tend to rely on these assumptions without recognizing that they have adopted them [1,2]. In addition, researchers begin to notice that there might be insufficient evidence to include some variables in clinical composite measures. Race and ethnicity are often considered in clinical composite measures to guide clinical practice [7]. However, some clinical algorithms may assign excessive weights to race and lead to inappropriate care for certain racial groups [7]. One recent study found that the symptoms used to diagnose mood disorders were not selected based on epidemiological evidence [5]. Another recent finding is that some of the frailty symptoms that researchers chose and used to diagnose frailty may be in fact not associated with frailty at all [9].

Index mining and syndrome mining

These problems embedded in some composite measures were recently identified using index mining and syndrome mining strategies that use systematic and comprehensive methods to aggregate information from a number of input variables [1,11]. Index mining has been developed when high-dimensional data are increasingly used in medical care and various statistical tools are introduced to create complicated measures for clinical use [12]. Index mining aims to use systematic approaches that can be applied to high-dimensional data (>1,900 variables) for discovering novel measures [12] or used to assess large numbers of newly derived indices or composite measures (976,676 measures examined) [11]. Index mining has been extended and actively used for recognizing syndromes that significantly predict mortality [11]. In practice, index or syndrome mining follows an established framework and begins with an assumption declaration, a selection of input variables, a choice of variable aggregation methods, and a selection of composite measures based on a set of criteria, such as mortality predictive power and interpretability [1,12].

Composite measure development framework

In a previous study, we were the first to identify the need to scrutinize composite measures and proposed a composite measure development framework based on the analyses of the Medical Expenditure Panel Survey (MEPS) data for principal component analysis (PCA) [4,12-14] and network analysis [15]. The MEPS participants were followed up for two years, and mortality was one of the outcomes [14]. We analyzed 1,989 MEPS variables using a weighted cumulative sample size of 4.5 billion Americans surveyed over 15 years [4]. Based on the PCA, input variables were assigned with mostly unequal weights to generate ‎orthogonal components [4]. A total of 134,689 novel composite measures were generated based on the input variables weighted by PCA loadings, and we tested them for their predictive power regarding mortality [4]. Based on this experience of creating, selecting, and assessing innovative composite measures, we proposed a research framework to help researchers understand the steps for mining innovative measures [4].

Despite the existence of a framework to develop composite measures, researchers have continued to use problematic composite measures in their latest research projects, particularly the recent studies involving problematic frailty measures [9] and mental illness diagnoses based on the same problematic assumptions [5]. The consequences of neglecting these problematic assumptions are significant. The diagnosis of frailty has failed to establish causal relationships with some of the frailty symptoms that are used to diagnose frailty [9]. One of the reasons that mental illness diagnoses are called wrong is that health professionals impose weighting schemes on mental symptoms that public members disagree with [5].

A comprehensive guide for reporting and critically assessing composite measures is needed. Based on our pioneering work in examining the assumptions of composite measures [1], a causation review of frailty [9], our systematic mining of new composite measures [1,11], and our statistical interpretation of composite measures [12,13,16], we aim to demonstrate a reporting guide and critical appraisal tool that researchers can use to evaluate innovative composite measures and readers can apply to assess published composite measures.

## Materials and methods

Development of the reporting guide and appraisal tool

This reporting and appraisal tool is applicable to composite measures that integrate information from multiple input variables, which are used to represent or symbolize certain theories or concepts. Without relevant precedents, the objectives of this tool include raising awareness of the assumptions of composite measures, demonstrating an evidence-based approach to developing composite measures, and providing a template for understanding these measures and for evaluating their strengths and weaknesses.

We developed this reporting and assessment tool based on evidence and the consensus of experts pioneering research on index mining and syndrome mining [1,11]. Initially, we designed the abovementioned development framework for composite measures [1], and then, we tested and revised it based on several composite measures commonly used in medical research, such as frailty indices, body mass index [1], three mental illness diagnoses [5], and innovative indices mined for mortality prediction [11].

Validation with frailty indices and their alternatives

In a previously published study to validate the proposed framework [4], we reproduced and reviewed three frailty indices and BMI using the Health and Retirement Study data according to Cigolle et al.’s (2009) study [1,17]. The frailty indices were the sums of domain variables that might be derived from two to five input variables [1]. We identified several sources of bias variables regarding the frailty indices, which mostly were due to inappropriate data processing, such as variable categorization and data censoring, while creating intermediate variables [1]. We identified bias variables and residuals of the domain variables that could not be explained by the input variables and retained them for analysis [1]. With the framework [4], we described in detail and critically appraised the assumptions used to generate the three frailty indices and BMI [1]. We discussed the methods used to select the input variables according to the theories that were proposed to support the use of the frailty indices [1]. For evaluation, we reproduced the bias variables induced by data processing and disclosed the implicit weighting schemes applied to the input variables by the indices [1]. We interpreted the indices with the input variables and bias variables using linear regression [1].

In another study, we also assessed other alternative composite measures that consisted of the symptoms used to diagnose frailty. There were 5,041 unequally weighted and 971,635 equally weighted four-item syndromes generated for assessment and mortality prediction [11]. The success rates to generate novel composite measures associated significantly with mortality were 55.5% and 99.3% for unequally and equally weighted measures, respectively [11]. The inclusion of the cognitive impairment variable was the key factor for creating the composite measures that had the largest effect sizes for mortality prediction [11]. The effect size of cognitive impairment alone was larger than those of the significant composite measures with the largest effect sizes [11]. Mathematically and empirically, composite measures are less likely to predict mortality better than their input variables [1,11].

Bias variables in the diagnoses of three mental illnesses

In previous studies, we found that the diagnoses of three mental illnesses were composite measures that had problems similar to other composite measures [5,8]. We reviewed the following three mental illness diagnoses: major depressive episodes, dysthymic disorder, and manic episodes [8]. Using the same framework [4], we identified that the undeclared assumptions in the diagnoses included implicit weighting schemes that distorted the importance of input symptoms, a disregard for the consequences of aggregating correlated or opposite variables, implicit upper limits of the prevalence of the diagnosis, and a lack of continuity of mental symptoms [8]. Given the input symptoms of similar prevalence, dysthymic disorder was more prevalent than the other two, major depressive episodes were less prevalent, and the diagnoses were less prevalent than the input symptoms [8]. The diagnoses were not well linked to the objectives of the diagnoses because certain input symptoms explained the diagnoses better than expected, and bias variables due to data processing were introduced into the diagnoses [8]. When the public was engaged and asked about the assumed relationships between mental illness diagnoses and their symptoms, they did not agree with all the assumptions, and we concluded that mental illness diagnoses were wrong for imposing arbitrary assumptions that lacked evidence to support [5].

Consensus on the questions in the tool

Based on the findings and the limitations of the composite measures we identified with the framework, we formulated questions to assess and use composite measures. Based on the validation, we discussed the identified issues and revised the framework [4] that we originally proposed for composite measure development. There were three rounds of question selection and revision. In the first round, YSC, HCW, CJW, and WCC first drafted a 22-question version based on the framework previously published [4]. In the second round, the four authors tried the questions using published composite measures, including frailty [1] and mental illness diagnoses [8], and assessed the time required for this tool. Three questions related to publication status and study design were removed based on consensus. In the third round, we invited clinical experts to form an expert panel to access this tool (HTH, LCT, YPC, and YCL). This panel reviewed the identified issues and considered other aspects that might have been neglected in previous studies [1,8]. The expert panel decided not to remove any questions and reached a consensus on the items to be used by the reporting and assessment tool. This panel also reviewed the notes to this tool and revised the texts to better describe the purposes and usefulness of the questions in this tool. All authors of the present study reviewed the tool and reached a consensus for finalizing a guide that researchers could use to develop innovative composite measures and that readers could use to assess published composite measures. While we continued reviewing other composite measures, we planned to revise and reassess this tool in the future.

## Results

This tool is specialized for the development and critical appraisal of composite measures, regardless of their contexts, and aims to provide further insight into the scientific rigor of the composite measures under evaluation. This tool should be used with relevant reporting guides or appraisal tools for risks of bias and does not replace the process of obtaining study approval, such as ethics review or trial registration.

Domains for reporting and critical appraisal

Our reporting guide and critical appraisal tool included 19 questions across seven domains. We based the questions on an initial assessment framework that researchers should consider while mining innovative composite measures [4]. For the questions applicable to the composite measures of interest, “yes,” “partial yes,” and “no” were valid answers. The questions for rating are listed in Table 1. The instruction for using the rating results was provided at the end of the Results.

**Table 1:** Domains and criteria to report or appraise the quality of composite measures

Reporting and appraisal criteria	Description and rating
Domain A: Candidate variable selection	
1. Were the candidate variables selected based on theories or evidence?	For yes:
	( ) The authors described the evidence or theories used to search for the candidate variables.
	For partial yes:
	( ) The authors declared the candidate variables were from data sets and theories without providing details.
	For no:
	( ) The authors did not provide enough information for evaluation.
2. Did the criteria for including or excluding the candidate variables match the theories or evidence?	For yes:
	( ) The variable inclusion and exclusion criteria were linked to the theories or evidence with sufficient details.
	AND
	( ) The reasons for inclusion and exclusion were specified for all candidate variables.
	For partial yes:
	( ) The authors linked the variable inclusion and exclusion criteria to the theories or evidence without a clear explanation.
	OR
	( ) Only the numbers of the candidate variables included or excluded were reported.
	For no:
	( ) The authors did not provide enough information for evaluation.
Domain B: Variable inclusion and assumption declaration	
3. Were the methods to assess the importance of the input variables declared explicitly with details?	For yes:
	( ) The authors declared the assumptions about the relationships between the input variables and the methods used to assess the assumptions were described.
	For partial yes:
	( ) The authors did not declare the attempts to investigate relationships between the input variables but reported assumptions identified in index mining literature, such as equal weights assigned to input variables.
	For no:
	( ) The authors did not provide enough information for evaluation.
4. Were the input variables appropriate for aggregation?	For yes:
	( ) The authors provided the evidence or used methods to show that aggregating input variables would not be problematic for issues, such as summing opposite variables.
	For partial yes:
	( ) The authors did not provide evidence or pilot studies that showed the input variables were appropriate for aggregation. However, a statement was provided regarding the assessment of the adequacy of variable aggregation.
	For no:
	( ) The authors did not provide enough information for evaluation.
5. When input variables were aggregated into composite measures, it was assumed that the input variables shared a common regression coefficient for outcome prediction. Was this assumption declared?	For yes:
	( ) The authors declared the assumptions about the relationships between the input variables when the composite measure was used for outcome prediction.
	For partial yes:
	( ) The authors did not declare the assumptions but did not recommend the composite measure for outcome prediction.
	For no:
	( ) The authors did not provide enough information for evaluation.
6. Were there other assumptions about variable selection and creation?	For yes:
	( ) The authors described other assumptions or declared no other assumptions.
	For no:
	( ) The authors did not provide enough information for evaluation.
Domain C: Data processing	
7. Was the information on missing data reported?	For yes:
	( ) The authors listed the information on missing data, such as the proportions of missingness for the input variables.
	For partial yes:
	( ) The authors reported minimal missingness without specifying details.
	For no:
	( ) The authors did not provide enough information for evaluation.
8. Were the methods for missing data reported and justified?	For yes:
	( ) The authors described and justified the methods for missing data.
	For partial yes:
	( ) The authors mentioned the methods for missing data.
	For no:
	( ) The authors did not provide enough information for evaluation.
9. Were the bias variables or residual variables created after each step of data processing?	For yes:
	( ) The authors listed information on the input and derived variables, and they also defined and described in detail the residuals of the derived variables that could not be explained by the input variables.
	For partial yes:
	( ) The authors reported on the creation of the bias variables or residual variables, similar to the approach taken by Chao et al. (2018) [1].
	For no:
	( ) The authors did not provide enough information for evaluation.
Domain D: Weighting schemes or methods to aggregate information	
10. Was the design of the weighting scheme reported and justified?	For yes:
	The authors declared the weighting scheme after
	( ) assessing the relationships between the candidate variables,
	AND
	( ) justifying the adoption of the input variables,
	AND
	( ) describing the methods to generate weights.
	For partial yes:
	The authors declared the weighting scheme after
	( ) assessing the relationships between the candidate variables
	OR
	( ) justifying the adoption of the input variables
	OR
	( ) describing the methods to generate weights.
	For no:
	( ) The authors did not provide enough information for evaluation.
11. Did the weighting schemes violate the declared assumptions?	For yes:
	( ) The authors described the weighting scheme and its relationships with the assumptions used for screening candidate variables or other assumptions required for index mining.
	For partial yes:
	( ) The authors declared the weighting scheme matched the assumptions listed in the screening of candidate variables.
	For no:
	( ) The authors did not provide enough information for evaluation.
Domain E: Applicability of the composite measure	
12. Were there other assumptions about the interpretation and usage of the composite measure?	For yes:
	( ) The authors defined the eligible populations and the occasions for the use of the composite measure. The composite measure also was tested in ineligible populations and on other occasions for verification.
	OR
	( ) The authors declared that no other assumptions were used.
	For partial yes:
	( ) The authors defined the eligible populations and the occasions for the use of the composite measure without clear justification.
	For no:
	( ) The authors did not provide enough information for evaluation.
Domain F: Composite measure interpretation	
13. Did the authors report the proportions of the composite measure variances explained by the input variables?	For yes:
	( ) The authors reported the proportions of the composite measure variances that could be explained by the individual input variables and justified the existence of bias variables or residuals if applicable.
	For partial yes:
	( ) The authors reported the proportions of the composite measure variances that could be explained by the input variables.
	For no:
	( ) The authors did not provide enough information for evaluation.
14. Did the authors report the proportions of the composite measure variances explained by the bias variables?	For yes:
	( ) The authors declared no bias variable was introduced by the data processing or information aggregation
	OR
	( ) The authors reported the proportions of the composite measure variances that could be explained by the bias variables.
	For no:
	( ) The authors did not provide enough information for evaluation.
15. Were composite measures well interpreted with input variables using linear methods?	For yes:
	( ) The proportions of the composite measure variances that could not be explained by the input variables were acceptable for constructing measures.
	For partial yes:
	( ) The proportions of the composite measure variances that could not be explained by the input variables or other assessment measures were claimed to be acceptable without justification.
	For no:
	( ) The authors did not provide enough information for evaluation.
16. Did the weighting scheme or aggregation methods match the interpretation?	For yes:
	There was enough information to confirm
	( ) that the composite measure could be interpreted with the input variables,
	AND
	( ) that the difference in the weighting scheme and the regression coefficients of the input variables regarding composite measure interpretation was acceptable,
	AND
	( ) that the residuals or bias variables of the composite measures were expected or essential for index mining.
	For partial yes:
	The authors declared
	( ) that the composite measure could be interpreted with the input variables,
	OR
	( ) that the differences in the weighting scheme and the regression coefficients of the input variables regarding composite measure interpretation were acceptable,
	OR
	( ) that the residuals or bias variables of the composite measure were expected or essential for index mining.
	For no:
	( ) The authors did not provide enough information for evaluation.
17. Were other techniques used to interpret the composite measure if linear approximation was not applicable?	For yes:
	There was enough information to confirm
	( ) that the composite measure could be interpreted with the input variables using designated methods.
	For partial yes:
	The authors declared
	( ) that the composite measure could be interpreted with the input variables using designated methods.
	For no:
	( ) The authors did not provide enough information for evaluation.
Domain G: Recommendations on the use of composite measures	
18. Were there certain populations eligible for the composite measure?	For yes:
	( ) The authors clearly stated the populations to which the composite measure can be applied, and evidence existed to show that the composite measure was sensitive to the target populations and had acceptable specificity to other populations.
	For no:
	( ) The authors did not provide enough information for evaluation.
19. Was the composite measure recommended for use as an outcome or predictor, and for what reasons?	For yes:
	( ) There was enough information to support the authors’ recommendation about using the composite measure as an outcome or predictor.
	For partial yes:
	( ) The authors’ recommendation about using the composite measure as an outcome or predictor can be partly supported by their findings.
	For no:
	( ) The authors did not provide enough information for evaluation.

Domain A: Candidate Variable Selection

Were the candidate variables selected based on theories or evidence?: Theory or evidence should be used to identify the candidate variables [1]. The desired characteristics of the candidate variables should be defined a priori. The statistical significance (p values) of the candidate variables for predicting outcomes should not be the sole justification for variable selection [1,11]. Clinical relevance, effect sizes, data sources, measurement validity, and reliability should be described to support variable selection. When possible, biological or pathological evidence should be illustrated to assess the generalizability and applicability of the desired measures in similar populations. A recent study that used syndrome mining concluded that the success rate of creating statistically significant syndromes for mortality prediction by summing any four age-related variables is greater than 99% [11]. When a composite measure is developed based on statistical significance, many other statistically significant alternatives could be identified using index mining.

Did the criteria for including or excluding the candidate variables match the theories or evidence?: Variable inclusion and exclusion criteria should be well described and linked to theories or evidence [1]. It is important to demonstrate that each of the variable inclusion and exclusion criteria is linked to theories or evidence. The description of the candidate variables should be clear and correspond to the selection criteria. When input variables are not selected based on evidence, composite measures may fail to demonstrate a significant association with the input variables [9].

Domain B: Variable Inclusion and Assumption Declaration

Were the methods to assess the importance of the input variables declared explicitly with details?: Some of the candidate variables can be selected and used as input variables to form composite measures. The importance of the input variables relative to other eligible variables needs to be declared or assessed [1,5]. Input variables are often assumed to be equally important and are summed to generate a composite measure [1,18]. For certain composite measures, input variables are assumed to interact with each other and are multiplied to generate a measure [1]. Assumptions about the importance or interaction of variables need to be documented here and examined later (Question 11).

Were the input variables appropriate for aggregation?: Some variables should not be summed or aggregated for a variety of reasons. For example, the input variables might be redundant or conflicting due to incompatible theories or measurement scales [10]. Mathematically, the sum of input variables might not be more meaningful than single input variables because these single variables are highly correlated or completely opposite [10]. Occasionally, input variables are independent of or orthogonal to each other [10]. The rationale to sum uncorrelated or unrelated variables into single indices needs to be elaborated. These issues need to be considered before mining composite measures. There were various methods to select features in data sets or evaluate the relationships between candidates or input variables, such as principal component analysis and correlation analysis [4,8,12]. An excessive inclusion of large numbers of input variables to generate single composite measures is likely to lead to overcomplicated measures that might not be different from those measures that use fewer input variables [1].

When input variables were aggregated into composite measures, it was assumed that the input variables shared a common regression coefficient for outcome prediction. Was this assumption declared?: When the composite measures are recommended for outcome prediction, this question needs to be answered. Aggregating input variables with equal or unequal weights into composite measures for outcome prediction in linear models is assuming that the importance of the input variables remains the same across all outcomes by imposing common regression coefficients on all input variables [1]. When the same composite measure is used to predict different outcomes in linear models, the regression coefficients of the composite measures vary, but the relative importance of the input variables remains the same because these input variables are combined [1,11]. If input variables are summed with equal weights to generate a composite measure for outcome prediction using linear models, it is assumed that these variables share a common regression coefficient [1,11]. When input variables are aggregated into composite measures using unequal weights or complicated algorithms, the importance of the input variables is not equal, but their importance relative to each other remains unchanged across all outcomes [1,11]. Mathematically, forcing input variables to share a common regression coefficient for outcome prediction is a restrictive assumption that researchers need to consider because single input variables are more likely to provide better model fit than composite measures regarding outcome prediction [1]. This assumption should be declared when reporting the methods, and it should be documented for a critical appraisal if the composite measure is recommended for outcome prediction.

Were there other assumptions about variable selection and creation?: In addition to the assumptions required for index mining mentioned above (Questions 3 and 5) [1,11], other assumptions may be used to create composite measures. For example, it may be assumed that the intermediate measures created by variable categorization or censoring are essential for generating composite measures [5]. Optimal ranges may be assumed for input variables and composite measures [1]. Another example is that ordinal variables may be assumed to be equivalent to interval variables [1]. These assumptions need to be declared and justified.

Domain C: Data Processing

Was the information on missing data reported?: Proportions of missing data and imputation methods are related to data quality and the adequacy of a designated data set. A lack of information disclosure can lead to poor reproducibility of composite measures [1]. Researchers should report any missing information in the variables.

Were the methods for missing data reported and justified?: Various means are available to deal with missing data. These methods need to be reported and justified. Multiple imputation, considered an option to deal with this problem, should be described in detail, which should include the variables on which the imputations were based [19]. A reporting guideline was published for authors to disclose the essential details in missing data imputations [19].

Were the bias variables or residual variables created after each step of data processing?: In many cases, composite measures are created from derived variables [1]. Certain data practices, especially categorization and censoring, introduce bias variables that cannot be explained by input variables [1]. The residuals, created with individual input variables, could be demonstrated as bias variables and be uncovered [1]. For example, when a continuous variable with values ranging from 0 to 1 is categorized into a binomial variable that contains only 0s and 1s, a bias variable can be derived by obtaining the residuals of the continuous variable that could not be explained by the binomial variable. These steps should be documented and clearly reported. To examine the role of bias variables (if applicable), these variables need to be retained as single variables after each step of data processing [1]. In some cases, bias variables could explain more than 71% of the variances of composite measures [1]. It is important to identify these bias variables for this question and evaluate how well they can explain the composite measures (Question 14).

Domain D: Weighting Schemes or Methods to Aggregate Information

Was the design of the weighting scheme reported and justified?: The method to aggregate information from the input variables could be to sum them with weights or to use nonlinear methods, such as multiplication [1]. How the variables are aggregated needs to be clearly stated and matched with the objectives of the composite measures. If a composite measure was created by summing input variables with given weights, the choice of equal or unequal weights should be described and justified [11]. When nonlinear methods were used to create novel composite measures, the use of these methods could be reported here and justified by demonstrating the interpretable relationships between input variables and the composite measures (Question 16) [1].

Did the weighting schemes violate the declared assumptions?: The weighting scheme or aggregation methods could be theory-based or data-driven, and they need to match the assumptions mentioned above regarding the relationships between the input variables [1], the assumptions used to search for the candidate variables, and the assumptions proposed in the theories.

Domain E: Applicability of the Composite Measure

Were there other assumptions about the interpretation and usage of the composite measure?: In addition to the assumptions required for mining novel composite measures, other assumptions may be required to apply these measures in research or the real world. For example, the assumption about the applicability of composite measures to certain populations needs to be declared. Often, frailty indices are assumed to be geriatric syndromes and are applied to those aged 65 years and over [1,17]. These assumptions should also be declared here and examined with recommendations on the use of composite measures (Questions 18 and 19). The applicable contexts need to be well described and tested with the following questions.

Domain F: Composite Measure Interpretation

Did the authors report the proportions of the composite measure variances explained by the input variables?: One basic method for interpreting composite measures is to linearly regress them (outcome of the regression analysis) on input variables (predictors of the regression analysis) [1]. Statistics are available that demonstrate the relationships between composite measures and input variables, particularly R-squared, p values, and regression coefficients [1]. The R-squared statistics represent the proportions of composite measure variances explained by the input variables [1,11]. However, the composite measures created might not be closely related to the input variables for several reasons, such as poor data processing and an inappropriate assessment of the relationships between the input variables [1]. As a result, some composite measures might be poorly associated with the input variables or could be explained by fewer input variables [1].

Did the authors report the proportions of the composite measure variances explained by the bias variables?: In some cases, bias variables might explain composite measures better than input variables [1]. This type of composite measure was not properly designed and could be identified with the bias variables [1]. After creating potential bias variables after each data processing step, the composite measures can be regressed by bias variables [1,8]. A clear statement should be made about the relationship, if any, between bias variables and composite measures.

Were composite measures well interpreted with input variables using linear methods?: When linear models are applicable, composite measures could be interpreted with input variables based on regression coefficients [8]. The coefficients of the input variables should be discussed in relation to theories, objectives, and variable selection. Statistics, such as p values and R-squared, could be used to support the use of the derived composite measures [1]. Researchers should provide a conclusion about the interpretability of composite measures with input variables using linear methods when applicable.

Did the weighting scheme or aggregation methods match the interpretation?: The weighing scheme is the relationships between the input variables that researchers aim to achieve when they sum input variables to create composite measures (Questions 10 and 11) [1,8]. However, while regressing the composite measures on the input variables, the regression coefficients of the input variables might not exactly match the weighting scheme for several reasons. First, the input variables might be highly correlated or redundant [1]. The sum of several highly correlated variables is not different from any one of them, particularly regarding outcome prediction [10]. Summing contradictory variables also leads to composite measures that cannot be properly interpreted [10]. Another reason that regression coefficients of the input variables might not exactly match the weighting scheme is that bias variables may be introduced during the data processing [1]. The authors should determine whether the differences between the regression coefficients and the weighting scheme were acceptable and for what reasons.

Were other techniques used to interpret the composite measure if linear approximation was not applicable?: In cases of binomial measures or measures of other formats, nonlinear or other methods may be more favorable for interpreting them [1]. For example, dichotomous measures could be interpreted with logit regression, and their predictive power could be evaluated with receiver operating characteristic curves or other statistics [16]. The key is to use the same statistics throughout the interpretation of composite measures with various input or bias variables [1].

Domain G: Recommendations on the Use of Composite Measures

Were there certain populations eligible for the composite measure?: Some measures are meaningful only for certain populations or circumstances, which should be specified. For example, when frailty is defined as a geriatric syndrome, most diagnostic criteria are applied to those aged 65 years and over [1]. However, when using the same criteria to diagnose frailty among those younger than the age threshold, the frailty criteria have poor specificity (falsely identifying cases among those not eligible for the diagnostic criteria) [1]. These populations and circumstances should be well described in the assumption statement (Question 6) and justified here.

Was the composite measure recommended for use as an outcome or predictor, and for what reasons?: After interpreting the composite measure, it is important to declare whether it is suitable for being an outcome for intervention or a variable for prediction. These reasons and rationales should be described. For example, composite measures that can be mostly explained by bias variables or fewer input variables should not be recommended for use as outcomes or predictors [1].

Overall rating

**Figure 1:**
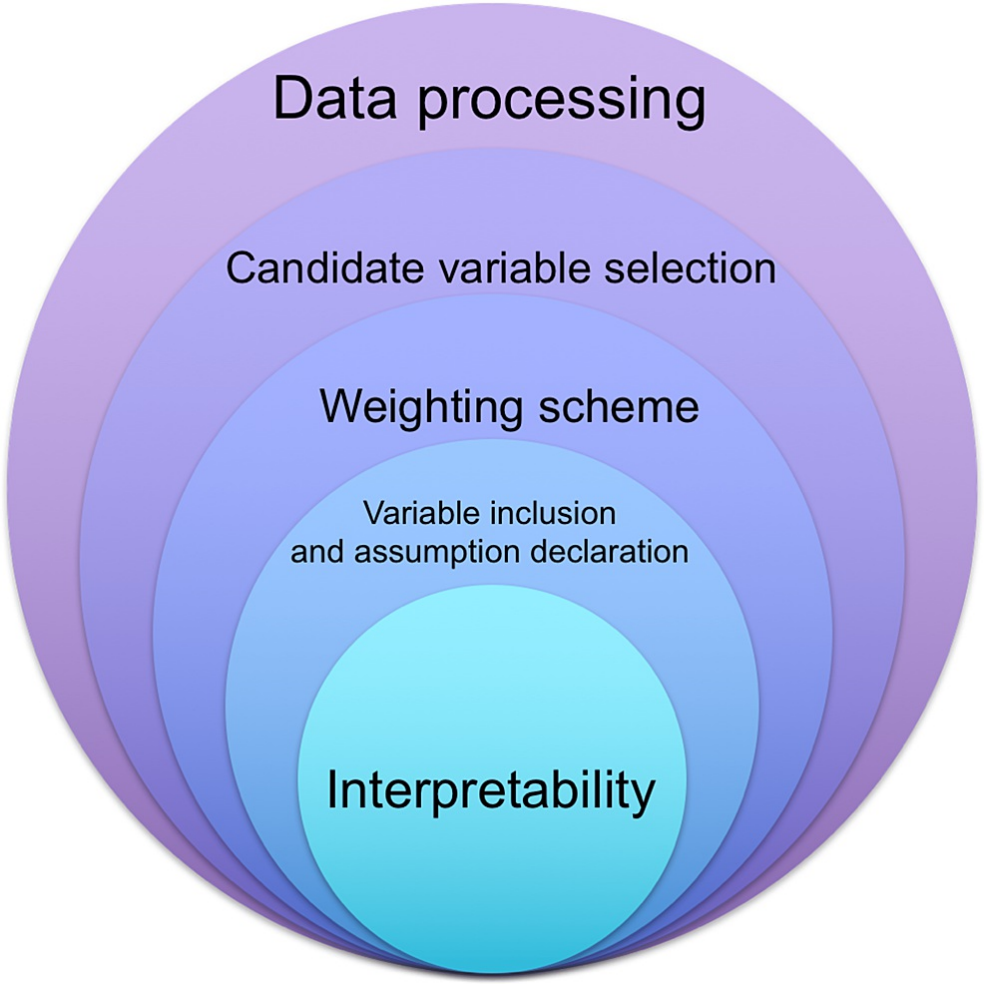
Hierarchy of the critical appraisal framework, the most important in central

From a hierarchical perspective (Figure 1), the interpretability of composite measures based on input variables is essential (Questions 13-17 in Domain F) [1]. If a composite measure could not be well approximated and interpreted by its input variables, this measure may be better explained by bias variables and subject to many problems, such as a lack of causal relationships with its input variables and a mismatch between the weighting scheme and observed regression coefficients. The next key domain is variable inclusion and assumption declaration (Questions 3-6 in Domain B). This domain helps depict the relationship between theories and input variables. In some cases, the theories may be abstract or too vague, so it is unclear how the input variables were selected [1,5,8]. The third important domain is the weighting scheme. Sometimes, researchers may not be aware of the assumptions related to index mining, so they apply equal weights regardless of context [18]. With the advances in index mining and increasing awareness, these assumptions need to be addressed and declared before input variables are aggregated. Other domains, such as candidate variable selection (Questions 1 and 2 in Domain A), data processing (Questions 7-9 in Domain C), methods to aggregate information (Questions 10 and 11 in Domain D), and recommendations on the use of the composite measures (Questions 18 and 19 in Domain G), are important, and they also are closely related to the interpretability and connection to the theories used. Our consensus is that readers are encouraged to first look at the interpretability of composite measures and the relationships between theories and derived measures. We recommend that researchers who are interested in creating their own composite measures report on all seven domains mentioned in our study with respect to their examination and research reproducibility.

## Discussion

This reporting and critical appraisal tool aims to use specific questions to guide authors and readers to appropriately report and examine innovative measures. Existing reporting or critical appraisal tools mostly focus on the validity of study designs [20] or predictive model development [21] or missing data imputation [19]. Sometimes, new measures are used in trials before they are found to be faulty or inferior to alternative measures [1]. If they are taken as outcomes, the patients recruited in such trials may experience unnecessary or even harmful interventions [22]. If they are used as outcome predictors, input variables are more likely to better predict outcomes [1]. Excessive use of composite measures for outcome prediction risks neglecting important input variables that have better predictive power [11,22]. The prediction models that use single input variables and better estimate health risks are likely to be unnoticed because of the overemphasis on novel composite measures [11,22]. One example is that cognitive impairment is the main reason why the frailty index defined by the Functional Domains Model had large effect sizes regarding mortality prediction, although cognitive impairment was not recognized for its leading role [22].

This reporting and assessment tool is a guide for researchers to think through the issues that they may encounter if they are interested in creating their own composite measures. In our studies, three of the most commonly used frailty indices [1] and three of the most common mental illness diagnoses [8] cannot be interpreted with the input variables in the ways described by their selection criteria. These composite measures are not connected to the theories that they are based on [1,8]. When novel composite measures are used for outcome prediction without scrutiny, researchers can be misled by their statistically significant p values and thus be motivated to publish these new measures [4,11]. However, it has been found that the success rate of obtaining statistically significant composite measures (p < 0.05) to predict mortality using age-related variables can be higher than 99%, regardless of the theories or concepts used to sum input variables [11]. When examining the composite measures that best predict mortality, their relatively large effect sizes are in fact driven by one single variable, cognitive deterioration [11]. We discovered that statistically significant composite measures with large effect sizes for mortality prediction could be quickly made by adding three variables with near-zero effect sizes with an input variable with a large effect size, cognitive deterioration [11]. These statistically significant measures can prevent readers and researchers from recognizing the importance of certain input variables, such as cognitive deterioration [11]. Researchers may design an intervention for these significant composite measures without recognizing the role of cognitive deterioration. Thus, a concern exists that less effective or even harmful interventions may be designed and introduced to patients [22]. In one recent study, mental illness diagnoses were called wrong for the undeclared assumptions and the disagreement in the interpretation of the diagnoses by professionals and public members [5].

The limited interpretability of three frailty indices and three mental illness diagnoses results from multiple causes, including a lack of input variable scrutiny, an absence of evidence-based variable selection criteria, and substandard data processing [1,5]. These issues can be identified with the items of the reporting and assessment tool of the present study. Readers may find it difficult to understand new composite measures based on a novel theoretical framework or statistical significance. Our tool provides a comprehensive list of items that readers can use to assess the validity and interpretability of new composite measures. Some measures may be defined differently by various research groups, such as metabolic syndrome and frailty syndrome [1]. Readers are encouraged to use the items in our tool to assess assumptions, the adequacy of variable selection, the connection between input variables and composite measures, and the magnitude of biases. Researchers are encouraged to think about these issues and try to avoid them [23].

With advances in and a wider application of data science and machine learning techniques, interpretability has become an issue of increasing importance. There are several approaches proposed for complicated measures or models. For example, the interpretability of machine learning can be investigated using various frameworks, such as a three-level approach by Doshi-Velez and Kim (2017) [24]. Two of the three levels require different levels of human interpretation, and one examines the level of complexity of the model [24]. There are explanation methods that aim to help humans understand model prediction [25]. These methods may require intensive human involvement and take time to understand the algorithms and model details. In contrast, R-squared has been widely used in linear models and recently applied to assess the interpretability of composite measures based on their input and bias variables [1,12]. Mental illness diagnoses are called wrong for the interpretability issues and arbitrary assumptions that some public members disagreed with [5]. In this tool, we suggested using well-established statistics, such as R-squared, regression coefficients, and pseudo-R-squared, to interpret composite measures by regressing composite measures on input variables (Question 13) and bias variables or measurement errors (Question 14). In our study, analyzing the interpretability of indices or composite measures in clinical trials, composite measures that are not likely well interpreted by their input variables, is associated with early termination of clinical trials [26].

It is important not to confuse this tool with other tools that aim to evaluate study design or risk of bias, such as the Consolidated Standards of Reporting Trials (CONSORT) Statement for randomized trials [27] and the Strengthening the Reporting of Observational Studies in Epidemiology (STROBE) statement for observational studies [20], or the framework to grade the quality of evidence, such as the Grading of Recommendations, Assessment, Development and Evaluations (GRADE) [28]. This tool can be used with these tools at the same time.

Strengths and limitations

The strengths of this tool include that it was developed based on evidence and the experience we obtained while reproducing and interpreting frailty indices, BMI [1], three mental illness diagnoses [5,8], and a large number of equally or unequally weighted composite measures based on age-related variables [11]. However, despite the evidence base, our tool also has limitations. The strategies of index mining and syndrome mining are new and require more research to uncover other assumptions and introduce new techniques to examine data processing, systematically search for candidate input variables, systematically examine novel composite measures, interpret nonlinear composite measures, and refine data aggregation methods. We are working on this limitation and will update our critical appraisal tool based on evidence.

## Conclusions

The current manuscript presents the first reporting and critical appraisal tool for innovative composite measures. Our tool can be used with the checklists for study design appraisal or predictive model reporting or missing data imputation and does not replace the process of obtaining study approval. The questions that our tool provides highlight the issues that need to be explicitly considered and that have been neglected previously. This tool is the first attempt to improve the interpretability and reproducibility of novel composite measures. This tool will be updated as advances in index mining and syndrome mining continue.
